# Genomic surveillance reveals low prevalence of livestock-associated methicillin-resistant *Staphylococcus aureus* in the East of England

**DOI:** 10.1038/s41598-017-07662-2

**Published:** 2017-08-07

**Authors:** Ewan M. Harrison, Francesc Coll, Michelle S. Toleman, Beth Blane, Nicholas M. Brown, M. Estee Török, Julian Parkhill, Sharon J. Peacock

**Affiliations:** 1 Department of Medicine, University of Cambridge, Box 157 Addenbrooke’s Hospital, Hills Road, Cambridge, CB2 0QQ United Kingdom; 2 London School of Hygiene & Tropical Medicine, Keppel Street, London, WC1E 7HT United Kingdom; 3 Wellcome Trust Sanger Institute, Wellcome Trust Genome Campus, Hinxton, Cambridge, United Kingdom; 4 Cambridge University Hospitals NHS Foundation Trust, Hills Road, Cambridge, CB2 0QQ United Kingdom; 5 Public Health England, Clinical Microbiology and Public Health Laboratory, Box 236, Addenbrooke’s Hospital, Hills Road, Cambridge, CB2 0QW United Kingdom

## Abstract

Livestock-associated methicillin-resistant *Staphylococcus aureus* (LA-MRSA) is an emerging problem in many parts of the world. LA-MRSA has been isolated previously from animals and humans in the United Kingdom (UK), but the prevalence is unknown. The aim of this study was to determine the prevalence and to describe the molecular epidemiology of LA-MRSA isolated in the East of England (broadly Cambridge and the surrounding area). We accessed whole genome sequence data for 2,283 MRSA isolates from 1,465 people identified during a 12-month prospective study between 2012 and 2013 conducted in the East of England, United Kingdom. This laboratory serves four hospitals and 75 general practices. We screened the collection for multilocus sequence types (STs) and for host specific resistance and virulence factors previously associated with LA-MRSA. We identified 13 putative LA-MRSA isolates from 12 individuals, giving an estimated prevalence of 0.82% (95% CI 0.47% to 1.43%). Twelve isolates were *mecC*-MRSA (ten CC130, one ST425 and one ST1943) and single isolate was ST398. Our data demonstrate a low burden of LA-MRSA in the East of England, but the detection of *mecC*-MRSA and ST398 indicates the need for vigilance. Genomic surveillance provides a mechanism to detect and track the emergence and spread of MRSA clones of human importance.

## Introduction

*Staphylococcus aureus* is a major cause of infection in hospitals and the community. The widespread dissemination of methicillin-resistant *S. aureus* (MRSA) adds complexity to the treatment of infection. *S. aureus* also colonises and infects wild and domestic animals and livestock, and in the latter causes economically important diseases such as mastitis in ruminants, and bumble foot in poultry. Several *S. aureus* multilocus sequence type (MLST) lineages are associated with animals, including clonal complex (CC)1 (livestock) CC5 (avian), CC130 (multi-host), CC133 (ruminants), CC151 (ruminants), CC425 (ruminants and wild mammals), and CC398 (livestock)^1–6^. Adaption of *S. aureus* for specific animal species can be mediated by as little as a single nucleotide polymorphism (SNP)^7^, while genetic decay^1^ and acquisition of mobile genetic elements encoding host specific virulence factors such as von Willebrand factor binding protein also play an important role^8, 9^. Resistance to tetracycline is common in livestock-associated (LA)-MRSA, and is associated with high usage of this drug in veterinary medicine^10^.

While animal-adapted *S. aureus* has been recognised for many years, more recently it has become clear that zoonotic transmission of *S. aureus* is relatively common^11^. The emergence and spread of LA-MRSA has been exemplified by CC398 MRSA, which is associated with pigs, poultry, cattle and horses^10^. Since its emergence in the early 2000′s, CC398 has gone on to cause a significant and increasing burden of human disease in a number of European countries^12, 13^. In the UK, there have been numerous reports of the presence of LA-MRSA in livestock and animal products^14–18^ and rare cases of human infections^3, 19^ caused by CC398 and several MRSA lineages (CC130, CC425 and CC1943) positive for a type XI SCC*mec* element which encodes the *mecC* ^20^. Two recent prevalence surveys targeting the livestock-associated *mecC*-MRSA in human and dairy cattle estimated that *mecC*-MRSA was present in 2.15% (95% confidence interval (CI) 1.17–3.91%) of English and Welsh dairy farms, and caused 0.45% (95% CI 0.24–0.85%) of human MRSA infections in England^16, 21^. However, no estimation of the total burden of LA-MRSA associated with human infection has been conducted in the UK. Here, we used an unbiased collection of MRSA genome sequences from a single year in Cambridgeshire to estimate the burden of LA-MRSA in a defined geographical region.

## Results

### Screening for LA-MRSA

We screened 2,282 MRSA isolates collected from 1,465 people during a prospective study conducted at the Public Health England Clinical Microbiology and Public Health Laboratory, Cambridge University Hospitals NHS Foundation Trust in Cambridge, UK between 18 April 2012 and 17 April 2013. We initially screened genome sequence data for putative LA-MRSA based on known genetic features of LA-MRSA, namely (i) the presence of *mecC* or (ii) specific MLST sequence types (ST) or CCs. This identified 15 isolates from 13 people (ST97 (n = 2), ST398 (n = 1), *mecC* positive (n = 12, which belonged to CC130 (n = 10), ST425 (n = 1), and 1 ST1943 (n = 1) (Table 1). We then expanded the screen to include virulence factors associated with LA-MRSA (LukM/F leukotoxin^22^, SaPI-carried von Willebrand binding protein (vWbp)^9^, the allele variant of *tst* (toxic shock syndrome toxin (TSST) found on the bovine staphylococcal pathogenicity island (SaPIbov)^23^ and the avian-associated prophages: *ϕ*Av1, φAvβ, and SaPIAv)^1^. This identified two isolates, both of which had been captured in the initial screen: an ST398 isolate (CBLA13 positive for the SaPI-carried *vwb*), and an ST1943 isolate (CBLA15 positive for the allelic variant of *tst* (Table 1). The minority of isolates (n = 3) were associated with clinical disease (skin and soft tissue infections in three people), the remainder being isolated from multisite screens (Table 1).

**Table 1:** Potential Livestock associated MRSA strains and associated data.

Isolate name	Sequence type	Clonal complex	Date	Source	Location^a^	Vitek antibiogram^b^	Resistance genes	Virulence genes	ERR accession	LA-MRSA
**CBLA1** ^**c**^	97	97	01/13	Screen swab	GP	PEN, FOX, OXA	*blaZ, mecA, cadD*	*scn, sak*	ERR715294	No
**CBLA2** ^**c**^	97	97	01/13	Screen swab	HOS	PEN, FOX, OXA	*blaZ, mecA, cadD*	*scn, sak*	ERR737075	No
CBLA3	1945	130	08/12	Screen swab	HOS	PEN, FOX	*blaZ*_*LGA251*_ *, mecC, arsB, arsC*	*scn, sak, edin-B, etd2*	ERR701929	Yes
CBLA4	1245	130	09/12	Screen swab	HOS	PEN, FOX	*blaZ*_*LGA251*_ *, mecC, arsB, arsC*	*edin-B, etd2*	ERR714935	Yes
CBLA5	CC130	130	02/13	Screen swab	HOS	PEN, FOX	*blaZ*_*LGA251*_ *, mecC, arsB, arsC, cadD*	*edin-B, etd2*	ERR737232	Yes
CBLA6	1245	130	03/13	SSTI	HOS	PEN, FOX, ERY	*msrA, smr* (*qacC), blaZ*_*LGA251*_ *, mecC, arsB, arsC*	*edin-B, etd2*	ERR737543	Yes
CBLA7	130	130	03/13	Screen swab	HOS	PEN, FOX	*blaZ*_*LGA251*_ *, mecC, arsB, arsC*	*edin-B, etd2*	ERR737555	Yes
**CBLA8** ^**d**^	1245	130	06/12	Screen swab	GP	PEN, FOX	*blaZ*_*LGA251*_ *, mecC, arsB, arsC*	*edin-B, etd2*	ERR737640	Yes
CBLA9	2574	130	05/12	SSTI	GP	PEN, FOX	*blaZ*_*LGA251*_ *, mecC, arsB, arsC*	*edin-B, etd2*	ERR204178	Yes
**CBLA10** ^**d**^	1245	130	05/12	Screen swab	HOS	PEN, FOX	*blaZ*_*LGA251*_ *, mecC, arsB, arsC*	*edin-B, etd2*	ERR211963	Yes
CBLA11	1245	130	06/12	Screen swab	HOS	PEN, FOX	*blaZ*_*LGA251*_ *, mecC, arsB, arsC*	*edin-B, etd2*	ERR212880	Yes
CBLA12	1245	130	06/12	Screen swab	HOS	PEN, FOX, OXA	*blaZ*_*LGA251*_ *, mecC, arsB, arsC*	*edin-B, etd2*	ERR212993	Yes
CBLA13	398	398	04/13	Screen swab	HOS	PEN, FOX, OXA, ERY, TET, CLI	*blaZ, mecA, ermC, tetM, tetK*,	*vwb (*SaPI encoded)	ERR715120	Yes
CBLA14	425	425	01/13	Screen swab	HOS	PEN, FOX,	*blaZ*_*LGA251*_ *, mecC, arsB, arsC, cadD*		ERR737103	Yes
CBLA15	1943	1943	03/13	SSTI	GP	PEN, FOX	*blaZ*_*LGA251*_ *, mecC, arsB, arsC, cadD*	*tst (*SaPIbov), *seg*, *sei*	ERR737480	Yes

^a^Location: HOS = Hospital, GP = General practitioner. ^b^Vitek antibiogram: Three letter codes indicate resistance to**:** PEN = Benzlpenicillin, FOX = Cefoxitin, OXA = Oxacillin, FUS = Fusidic acid, ERY = Erythromycin, TET = Tetracycline, CLI = Clindamycin. Bold text - ^**c**^denotes that CBLA1 and CBLA2 were isolated from the same patient. ^d^denotes that CBLA8 and CBLA10 were isolated from the same patient. SSTI = skin and soft tissue infection.

### Phylogenetic analyses of LA-MRSA

**Figure 1:**
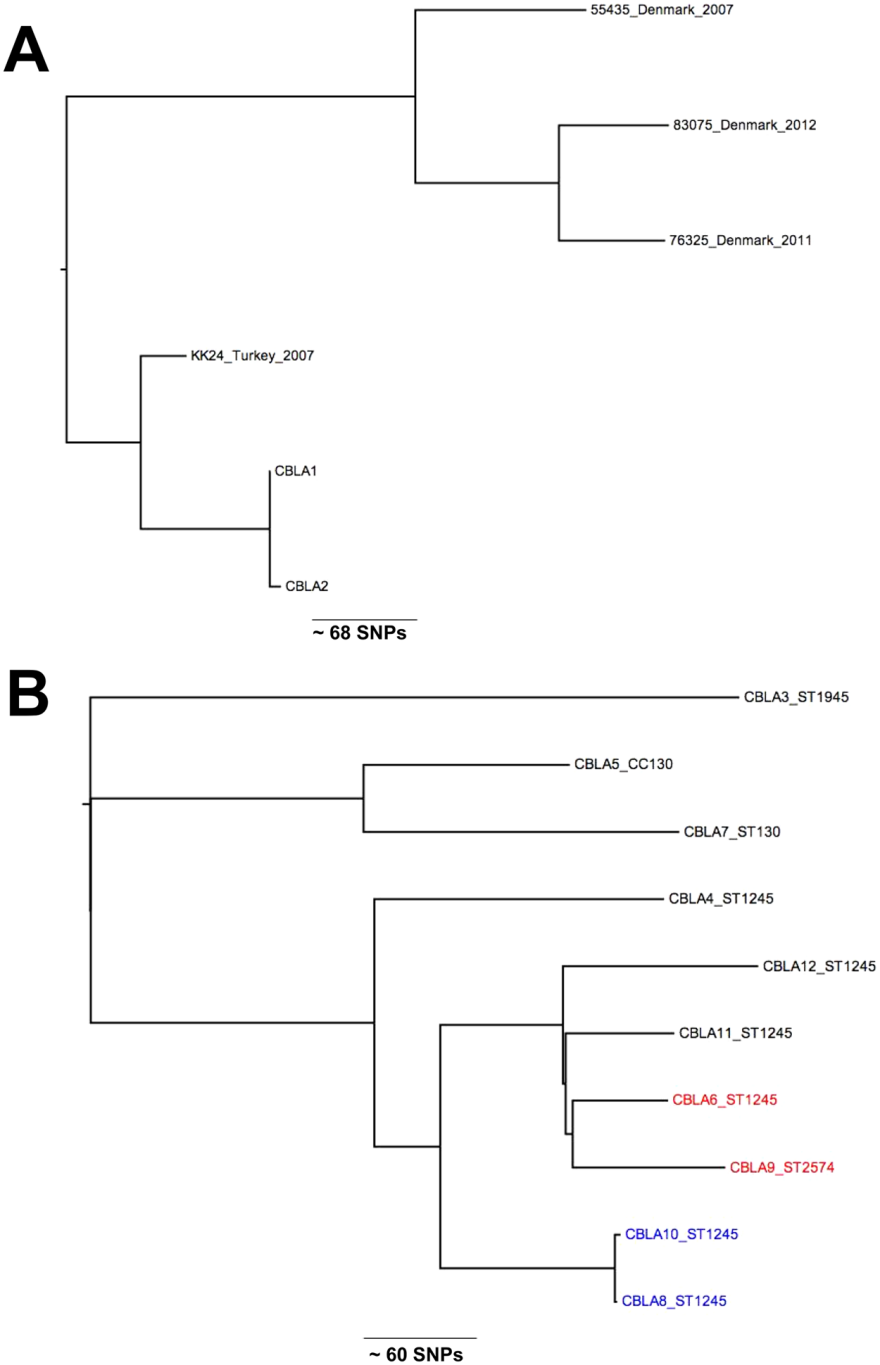
(**A**) Phylogenetic relationships between Cambridgeshire CC97 isolates and most closely related isolates from a global collection^10^. Figure shows an unrooted maximum likelihood tree generated from core genome single nucleotide polymorphisms. (**B**) Phylogenetic relationships between Cambridgeshire CC130 isolates. Figure shows an unrooted maximum likelihood tree generated from core genome single nucleotide polymorphisms. The two clinical isolates are highlighted in red. The two closely related isolates from the same patient are highlighted in blue.

*S. aureus* CC97 underwent a host jump from cattle into humans around 40 years ago^24^.To determine whether the two Cambridgeshire CC97 isolates (from a single individual) belonged to the cattle or human clade, we compared these with genomes of a global collection of 43 CC97 isolates^24^. A phylogenetic tree demonstrated that the two Cambridge ST97 isolates resided in the human clade A, and were most closely related to an isolate associated with a bloodstream infection in Turkey in 2007 (Fig. 1A)^24^. The host jump of CC97 into humans has been linked to the gain of a β-toxin-converting phage (φSa3) containing the human immune evasion cluster (IEC) of genes (staphylokinase (*sak*), staphylococcal complement inhibitor (*scn*), and chemotaxis inhibitory protein of *S. aureus* (*chp*)). The two Cambridgeshire CC97 isolates were positive for *sak* and *scn*, (IEC type E), providing further evidence of a human source (Table 1). We conclude that the Cambridgeshire ST97 isolates were not of a livestock origin, but rather a rare human adapted lineage of ST97 MRSA.

The genetic relatedness between the Cambridgeshire *mecC* ST425 isolate (CBLA14) and the ST425 reference genome (an English bovine isolate, LGA251) was defined based on SNPs in the core genome^3^. The two isolates differed by more than 400 core genome SNPs, indicating that they were distantly related. A phylogenetic tree of the ten CC130 *mecC*-MRSA isolates was reconstructed based on SNPs in the core genome compared with a reference. This showed clustering that was consistent with ST designation, although isolates belonging to the same ST were not closely related (>50 SNPs different) (Fig. 1B). The exception was two ST1245 isolates (CBLA8 and CBLA10) isolated from the same patient 19 days apart that differed by only four SNPs. In contrast to the rest of the *mecC*-MRSA isolates, the single ST1945 (CBLA3) was positive for *sak* and *scn*, (IEC type E) suggestive of a human origin. However, ST1945 isolates from wild mice and deer in Spain have also been found to be positive for IEC type E, suggesting this may be conserved trait of ST1945 isolates, irrespective of host^25, 26^.

To determine the genetic relatedness of the Cambridgeshire ST398 isolate (CBLA13) to those found elsewhere, we compared this with a global collection of 89 CC398 isolates^10^. A phylogenetic tree demonstrated that CBLA13 resided in the livestock-associated clade and was most closely related to three isolates with a porcine origin, in subclade IIa1ii from Austria and Slovenia (data not shown). This is consistent with a livestock source for CBLA13.

### Antimicrobial resistance

With one exception (CBLA12), *mecC*-MRSA isolates were resistant to cefoxitin, but susceptible to oxacillin based on VITEK testing, as reported previously (Table 1)^27^. Most *mecC*-MRSA isolates (11/12) were relatively susceptible, with resistance limited to β-lactam antibiotics out of the panel of agents tested. The exception (CBLA6) was resistant to erythromycin, associated with the presence of *msrA*, and also carried *smr (*formly *qacC)* that mediates resistance to quaternary ammonium compounds in antiseptics and disinfectants^28^. The single ST398 isolate (CBLA13) was resistant to erythromycin and tetracycline, which was associated with the presence of *ermC*, and *tetK* and *tetM* respectively. Tetracycline resistance is not unique to livestock-associated MRSA but is notable, as this is associated with livestock-associated ST398^10^.

## Discussion

Here, we report an unbiased assessment of the prevalence of LA-MRSA isolated by a single diagnostic microbiology laboratory in the East of England. By searching for lineages and virulence genes associated with animal MRSA we identified 13 people (15 isolates) positive for MRSA, with features associated previously with LA-MRSA. Detailed genome-level analysis supported a plausible link to livestock for 12 cases (13 isolates). The two exceptions (two isolates from the same case) were ST97, which were assigned to the human clade of a lineage that underwent a host-jump from cattle to humans around 40 years ago, and were therefore unlikely to be livestock-associated^24^. For the remaining 13 isolates (12 *mecC*-MRSA (CC130, ST425 and ST1943) and 1 ST398), the evidence suggests a likely livestock source, giving an overall prevalence rate of LA-MRSA in MRSA-positive people of 0.82% (95% CI 0.47% to 1.43%).

In the UK, ST398 has been detected in cattle, horses, pigs and pork meat, poultry, and in humans.^12, 14, 17, 19, 29–31^ The single ST398 isolate in this study clustered with the livestock-associated clade identified by Price and co-workers, and was most closely related to isolates from pigs in continental Europe^10^. Consistent with a livestock origin was the observation that the isolate carried a gene encoding a von Willebrand factor binding protein that mediates clotting of ruminant plasma, suggesting that this isolate might have ruminant source. This concurs with previous reports of ST398 in bovine milk in England^17^. However, *vwb* has also been found in three ST398 MRSA isolates from UK retail pork samples, suggesting this might be widespread in UK ST398 isolates^14^. The isolate was also resistant to tetracycline, which is also associated with LA ST398. We noted that the isolate harboured two different tetracycline resistance genes (*tetM* and *tetK)*, which has also been reported in Scottish Human CC398 isolates^19^. The acquisition of *tetK* as part of the staphylococcal cassette chromosome *mec* (SCC*mec*) type Vc element by *tetM* positive LA ST398 has been demonstrated to significantly increase fitness at sub-lethal concentrations of tetracycline^32^. The use of tetracycline in livestock is likely to be a driver for this and might have contributed to the success of SCC*mec* Vc-bearing LA-MRSA CC398^32^. In addition, the presence of the *czrC* gene encoding resistance to copper and zinc, which are added to animal feed, may have also contributed to the success of LA-MRSA CC398^34^. Although there are currently very low levels of ST398 in humans in the UK, its presence in a range of livestock species combined with the results of mathematical modelling suggests that once established in livestock populations, ST398 would be hard to eradicate from humans^33^. A 66% increase in human CC398 cases in Denmark between 2004–2011 was associated with a four-fold increase in the CC398 prevalence in Danish pigs^13^. Similar dramatic increases in ST398 prevalence have been reported in Germany^34^. Recent data has also highlighted the role of humans as the vector for transmission between livestock populations including between countries^35^. Further systematic sampling of livestock and livestock workers for ST398 in the UK is required to better understand its prevalence and epidemiology.

The majority of LA-MRSA isolates were *mecC*-MRSA, demonstrating that at least in this part of England, this is the dominant form of LA-MRSA. The prevalence in this study for *mecC*-MRSA of 0.75% (95% CI: 0.30% to 0.92%) is close to the 0.45% (95% CI 0.24%–0.85%) identified in a 2011–2012 multicentre English prevalence study, suggesting that prevalence is currently stable^21^. This is similar to a prevalence estimate from Denmark (0.5%)^36^ but higher than large studies from Belgium (0.18%)^37^, Germany (0, 0.09, and 0.06%)^38–40^, and Spain (0.04%)^41^, though some variation exists between studies from the same countries^42^. Both ST1245 isolates and its single locus variant ST2574 have been identified previously in Cambridge, in a multicentre English prevalence study, suggesting that these two clones are endemic in Cambridgeshire^21^. Importantly, ST1245 has also been isolated from bovine milk at two different locations in England^3^. Cambridgeshire is an area with low livestock density, but borders East Anglia where there is high density of pig and poultry production^43^. The predominance of CC130 in the Cambridgeshire LA-MRSA, a clone which is commonly isolated from sheep and cattle^3, 11, 44, 45^, suggests that the prevalence of CC130 might be higher in parts of the UK with higher sheep and cattle densities, or alternatively the reservoirs of *mecC*-MRSA are still not fully understood. Indeed, *mecC*-MRSA has recently been reported in pigs and pig workers in Denmark^46^. Five of the eight ST1245 isolates were detected in May and June (Table 1) when people are more likely to be outdoors and may come in contact with livestock, but this is underpowered and future studies are required to investigate this association in a robust manner.

In summary, our data demonstrate a low burden of LA-MRSA in the East of England, but the detection of *mecC*-MRSA and ST398 indicates the need for vigilance. As demonstrated here and elsewhere^47^, genomic surveillance provides a mechanism to detect and track the emergence of MRSA clones of human importance.

## Materials and Methods

### Study design

To understand the molecular epidemiology and transmission pathways in a healthcare network we conducted a prospective observational cohort study between April 2012 and April 2013. We identified all individuals with MRSA-positive samples processed by the Public Health England Clinical Microbiology and Public Health Laboratory, Cambridge University Hospitals NHS Foundation Trust in Cambridge, UK. The laboratory processes samples from four Cambridgeshire hospitals (Addenbrooke’s hospital (a large university teaching hospital), the Rosie hospital (a maternity hospital), Papworth hospital (a specialist cardiothoracic hospital) and Hinchingbrooke hospital (a district general hospital) and 75 general practices in the same geographic region (broadly the area around Cambridge). Samples were from multisite screening swabs from hospital patients on admission or during a hospital stay, or from clinical specimens from hospital patients or taken from patients in general practice. All cases with MRSA isolated at least once, from either screening swabs and/or clinical specimens, were included in the study. Clinical metadata and demographic information were collected from electronic and paper medical records. In accordance with national policy at this time, universal MRSA screening (a multi-site MRSA screen of all patients on hospital admission, and weekly MRSA screening of patients in critical care units) was conducted at all four hospitals throughout the study period.

### Ethics

The study protocol was approved by the National Research Ethics Service (ref: 11/EE/0499), the National Information Governance Board Ethics and Confidentiality Committee (ref: ECC 8–05(h)/2011), and the Cambridge University Hospitals NHS Foundation Trust Research and Development Department (ref: A092428).

### Microbiology methods

MRSA was isolated from screening samples by directly plating swabs onto Brilliance MRSA chromogenic medium (Oxoid, Basingstoke, UK), and from all other samples by plating onto Columbia Blood Agar (Oxoid, Basingstoke, UK). *S. aureus* was identified using a commercial latex agglutination kit (Pastorex Staph Plus, Bio Rad Laboratories, Hemel Hempstead, UK). Antimicrobial susceptibility was determined to a panel of antibiotics (benzylpenicillin, cefoxitin, oxacillin, ciprofloxacin, erythromycin, chloramphenicol, daptomycin, fusidic acid, gentamicin, linezolid, mupirocin, nitrofurantoin, rifampicin, teicoplanin, tetracycline, tigecycline, trimethoprim, vancomycin, clindamycin, and inducible resistance to clindamycin) using the VITEK 2 instrument (bioMerieux, Marcy l’Etoile, France). Antimicrobial susceptibility results were interpreted using the European Committee on Antimicrobial Susceptibility Testing (EUCAST) criteria^48^.

### Whole genome sequencing and bioinformatics analysis

Genomic DNA was extracted from MRSA isolates, libraries prepared and 150-bp paired end sequences determined on an Illumina HiSeq. 2000 as previously described^49^. Sequence data have been submitted to the European Nucleotide Archive (ENA) (www.ebi.ac.uk/ena) under the accession numbers listed in Table 1. Sequence data were assembled using a previously described pipeline^50^. Briefly, for each isolate the sequence reads were used to create multiple assemblies using VelvetOptimiser v2.2.5^51^ and Velvet v1.2^52^. The assemblies were improved by scaffolding the best N50 and contigs using SSPACE^53^ and sequence gaps filled using GapFiller^54^. Multilocus sequence types (MLST) were determined from the assemblies using MLST check (https://github.com/sanger-pathogens/mlst_check), which was used to compare the assembled genomes against the MLST database for *S. aureus* (http://pubmlst.org/saureus/). The presence of *S. aureus* virulence factors and antibiotic resistance genes were identified using BLAST against the assemblies. For phylogenetic analyses, sequence reads were mapped to a relevant reference genome (CC130 and ST425 isolates (strain LGA251, accession number FR821779), ST97 (strain MW2, accession number BA000033), ST398 (strain S0385, accession number AM990992)) using SMALT (http://www.sanger.ac.uk/science/tools/smalt-0) using the default settings to identify single nucleotide polymorphisms (SNPs). SNPs located in mobile genetic elements were removed and a maximum likelihood tree created using RAxML using the default settings and 100 bootstrap replicates^55^. For the ST398 phylogeny the large block of ST8 recombination present in ST398 (S0385 genomic locations: 12252 to 135180) was also removed from the ST398 alignment.
